# Supplementary material for: The Small RNA Universe of Capitella teleta
Source: Front Mol Biosci. 2022 Feb 25;9:802814. doi: 10.3389/fmolb.2022.802814 (PMC8915122; doi:10.3389/fmolb.2022.802814)
Supplement: Supplementary file 1 [file DataSheet1.ZIP › Supplement/known/CAPTEscaffold_133_10017.pdf]

Provisional ID	:	CAPTEscaffold_133_10017
Score total	:	274568.7
Score for star read(s)	:	3.9
Score for read counts	:	274558.3
Score for mfe	:	2
Score for randfold	:	1.6
Score for cons. seed	:	3
Total read count	:	538546
Mature read count	:	532453
Loop read count	:	0
Star read count	:	6093

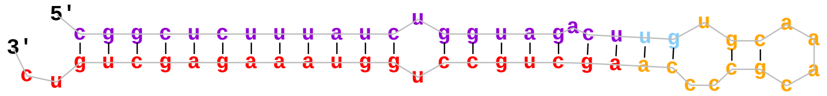

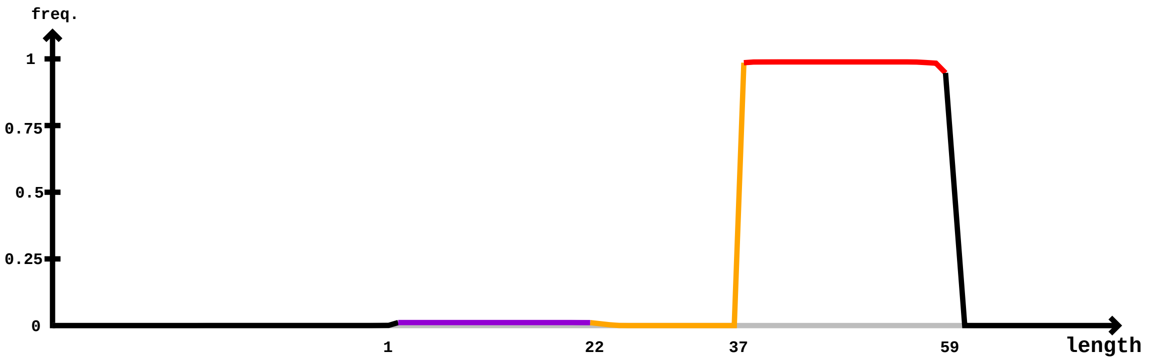

Star Mature

```
5'- cuccugugcucacgucuccugguugaggacaguacggcucuuuaucugguagacuugugcaaacgccccaagcugccugguaaagagcugucucgucgcaacccuucuc  -3'  obs
    cuccugugcucacgucuccugguugaggacaguacggcucuuuaucugguagacuugugcaaacgccccaagcugccugguaaagagcugucucgucgcaacccuucuc        exp
    cuccugugcucacgucuccugguugaggacaguacggcucuuuaucugguagacuugugcaaacgccccaagcugccugguaaagagcugucucgucgcaacccuucuc        known
    ....................(((((..(((((.(((((((((((((.(((((.((((.((....))..))))))))).))))))))))))))).))).))))).......  reads  mm  sample

    ............cgucuccugguugaggacagua...........................................................................  3     0   seq
    .............gucuccugguugaggacag.............................................................................  1     0   seq
    .............Uucuccugguugaggacagua...........................................................................  1     1   seq
    .............gucuccugguugaggacagua...........................................................................  4     0   seq
    ..............ucuccugguugaggacag.............................................................................  2     0   seq
    ..............ucuccugguugaggacagu............................................................................  4     0   seq
    ..............ucuccugguugaggacagua...........................................................................  52    0   seq
    ................................Nacggcucuuuaucugguagac.......................................................  1     1   seq
    ................................uacggcucuuuaucugguagac.......................................................  1     0   seq
    ................................uacggcucuuuaucugguagacu......................................................  96    0   seq
    .................................acggcucuuuaucugguaga........................................................  87    0   seq
    .................................acggcucuuuaucugguagac.......................................................  4     0   seq
    .................................acggcucuuuauGgguagacu......................................................  1     1   seq
    .................................Ccggcucuuuaucugguagacu......................................................  6     1   seq
    .................................acggcucuuuaucugguagacu......................................................  53    0   seq
    .................................Gcggcucuuuaucugguagacu......................................................  1     1   seq
    .................................acggcucuuAaucugguagacu......................................................  1     1   seq
    .................................acggcuUuuuaucugguagacuu.....................................................  1     1   seq
    .................................acggcucuuuaucugguagacuu.....................................................  14    0   seq
    ..................................cggcucuuuaucugguaga........................................................  2     0   seq
    ..................................cggcucuuuaucugguagac.......................................................  64    0   seq
    ..................................cggcucuuuaucuAguagac.......................................................  1     1   seq
    ..................................cggcGcuuuaucugguagacu......................................................  2     1   seq
    ..................................cggcucuuuaucuggAagacu......................................................  1     1   seq
    ..................................cggcucuuuaucGgguagacu......................................................  1     1   seq
    ..................................Uggcucuuuaucugguagacu......................................................  1     1   seq
    ..................................cggcucAuuaucugguagacu......................................................  2     1   seq
    ..................................cggcucuuuaucugguagacG......................................................  6     1   seq
    ..................................cggcAcuuuaucugguagacu......................................................  2     1   seq
    ..................................cggcucuuuaucuAguagacu......................................................  2     1   seq
    ..................................cggcucuuuaucugguagacu......................................................  3672  0   seq
```

Star Mature

```
cuccugugcucacgucuccugguugaggacaguacggcucuuuaucugguagacuugugcaaacgccccaagcugccugguaaagagcugucucgucgcaacccuucuc
..................................cggcucuuuaucugAuagacu......................................................  1     1  seq
..................................cggcucuuuaucugguagacC......................................................  12    1  seq
..................................cAgcucuuuaucugguagacu......................................................  1     1  seq
..................................cggcucuuuaucugguagacA......................................................  1     1  seq
..................................cggcucuAuaucugguagacu......................................................  2     1  seq
..................................cggcucuuuaucugguGgacu......................................................  1     1  seq
..................................cggcucuuuaucugguagaUu......................................................  1     1  seq
..................................cgUcucuuuaucugguagacu......................................................  1     1  seq
..................................cggcuAuuuaucugguagacu......................................................  1     1  seq
..................................cggcCcuuuaucugguagacu......................................................  1     1  seq
..................................cggcucuuuaucuggCagacu......................................................  2     1  seq
..................................Gggcucuuuaucugguagacu......................................................  1     1  seq
..................................cUgcucuuuaucugguagacu......................................................  1     1  seq
..................................Uggcucuuuaucugguagacuu.....................................................  16    1  seq
..................................cggcucuuuaucugguagacuu.....................................................  366   0  seq
..................................cggcAcuuuaucugguagacuu.....................................................  1     1  seq
..................................cggcucuuuaucugguagacuA.....................................................  4     1  seq
..................................cAgcucuuuaucugguagacuu.....................................................  1     1  seq
..................................cggcucuuuauAugguagacuu.....................................................  1     1  seq
..................................cggcucCuuaucugguagacuug....................................................  1     1  seq
..................................Nggcucuuuaucugguagacuug....................................................  1     1  seq
..................................cggcucuuuaucuAguagacuug....................................................  4     1  seq
..................................cggcucuuuaucugguagacuuU....................................................  11    1  seq
..................................cggcucuuuaucugguaAacuug....................................................  1     1  seq
..................................cggcucuuuaucugguagacuGg....................................................  1     1  seq
..................................cggcAcuuuaucugguagacuug....................................................  1     1  seq
..................................cggcuUuuuaucugguagacuug....................................................  1     1  seq
..................................cggcucuuuaucugguagaAuug....................................................  2     1  seq
..................................cggcucuuuaucugguGgacuug....................................................  1     1  seq
..................................cAgcucuuuaucugguagacuug....................................................  1     1  seq
..................................cggcucuuuUucugguagacuug....................................................  1     1  seq
..................................cggcucuuuaucugguagacuug....................................................  1430  0  seq
..................................cggcucuuuaAcugguagacuug....................................................  2     1  seq
..................................cggcuAuuuaucugguagacuug....................................................  1     1  seq
..................................cggcucuuuaucugguUgacuug....................................................  1     1  seq
..................................cggcucuuuaucugguagacuuC....................................................  2     1  seq
..................................cggcucuCuaucugguagacuug....................................................  1     1  seq
..................................cggcucAuuaucugguagacuug....................................................  1     1  seq
..................................cggcucuuuaucugguagacuuA....................................................  2     1  seq
..................................cggcucuuuaucuggAagacuug....................................................  1     1  seq
..................................cggcucuuuaucugguagGcuug....................................................  3     1  seq
..................................cggcucuuCaucugguagacuug....................................................  1     1  seq
..................................cggcucuuuGucugguagacuug....................................................  1     1  seq
..................................cggcucuuuauAugguagacuug....................................................  1     1  seq
..................................cggcucuuuaucugguagacuugu...................................................  131   0  seq
..................................cgCcucuuuaucugguagacuugu...................................................  1     1  seq
..................................cggcucuuuaucugguagacuugA...................................................  16    1  seq
..................................cggcucuuuaucugguagacuuguA..................................................  1     1  seq
..................................cggcucuuuaucugguagacuuguU..................................................  3     1  seq
...................................ggcucuuuaucugguagacu......................................................  12    0  seq
...................................ggcucuuuaucugguagacuu.....................................................  1     0  seq
...................................ggcucuuuaucugguagacuug....................................................  7     0  seq
...................................ggcucuuuaucugguagacuAg....................................................  1     1  seq
....................................gcucuuuaucugguagacu......................................................  6     0  seq
.....................................cucuuuaucugguagacu......................................................  4     0  seq
....................................................................Aaagcugccugguaaagagcuguc.................  2     1  seq
.....................................................................aagcugccugguaaagagcugu..................  2     0  seq
.....................................................................aagcugccugguaaagagcuguU.................  2     1  seq
.....................................................................Gagcugccugguaaagagcuguc.................  1     1  seq
.....................................................................aagcugccugguaaagagcuguc.................  1     0  seq
.....................................................................Cagcugccugguaaagagcuguc.................  10    1  seq
.....................................................................aagcugccugguaaagagcugucu................  2     0  seq
......................................................................agcugccuggAaaagagc.....................  1     1  seq
......................................................................agcAgccugguaaagagc.....................  1     1  seq
......................................................................agcugccCgguaaagagc.....................  1     1  seq
......................................................................agcugcAugguaaagagc.....................  1     1  seq
......................................................................Ggcugccugguaaagagc.....................  4     1  seq
......................................................................agcugccugAuaaagagc.....................  1     1  seq
......................................................................agcugccugguaaagagA.....................  1     1  seq
......................................................................agcugccugguaaagagc.....................  232   0  seq
```

Star **Mature**

```
cuccugugcucacgucuccugguugaggacaguacggcucuuuaucugguagacuugugcaaacgccccaagcugccugguaaagagcugucucgucgcaacccuucuc
......................................................................agcugccugguaaagagcG....................	8	1	seq
......................................................................agcugccugguaaagagcC....................	1	1	seq
......................................................................Ggcugccugguaaagagcu....................	12	1	seq
......................................................................agcuAccugguaaagagcu....................	1	1	seq
......................................................................agcAgccugguaaagagcu....................	1	1	seq
......................................................................agAugccugguaaagagcu....................	1	1	seq
......................................................................agcugccuCguaaagagcu....................	1	1	seq
......................................................................agcugccugguaGagagcu....................	2	1	seq
......................................................................agcugccugguaaGgagcu....................	1	1	seq
......................................................................agcugccugguaaagagcu....................	1036	0	seq
......................................................................agcugccuggAaaagagcu....................	1	1	seq
......................................................................agcugccuUguaaagagcu....................	1	1	seq
......................................................................agcugccugguaaagaCcu....................	1	1	seq
......................................................................agcugccugguaaagagcuA...................	2	1	seq
......................................................................Ggcugccugguaaagagcug...................	13	1	seq
......................................................................agcugccugguaaagagcuU...................	16	1	seq
......................................................................agcugccugguaaagGgcug...................	2	1	seq
......................................................................agcugccuggAaaagagcug...................	1	1	seq
......................................................................agcugccuAguaaagagcug...................	1	1	seq
......................................................................agcugccugguaaagagcug...................	1200	0	seq
......................................................................agcugccugguaaagagUug...................	1	1	seq
......................................................................agcugAcugguaaagagcug...................	1	1	seq
......................................................................agcugccugguaaagagcuC...................	3	1	seq
......................................................................agcugccugguaaagagcGg...................	3	1	seq
......................................................................agcugccugAuaaagagcug...................	1	1	seq
......................................................................agcugccCgguaaagagcug...................	1	1	seq
......................................................................agcugccugguaaagagAug...................	1	1	seq
......................................................................agcugccuggGaaagagcug...................	1	1	seq
......................................................................agcAgccugguaaagagcugu..................	12	1	seq
......................................................................agcugccuggCaaagagcugu..................	1	1	seq
......................................................................agcugccugCuaaagagcugu..................	3	1	seq
......................................................................agcugccugguaUagagcugu..................	2	1	seq
......................................................................agcugccugguaaagagcugu..................	19218	0	seq
......................................................................agcugcUugguaaagagcugu..................	2	1	seq
......................................................................agcugccugguaaGgagcugu..................	2	1	seq
......................................................................agAugccugguaaagagcugu..................	6	1	seq
......................................................................agcugccugguaaUgagcugu..................	1	1	seq
......................................................................agcugccugguaaagagcugA..................	9	1	seq
......................................................................agcuUccugguaaagagcugu..................	1	1	seq
......................................................................agcugccugguaGagagcugu..................	7	1	seq
......................................................................agcugccugUuaaagagcugu..................	1	1	seq
......................................................................agcugccuggAaaagagcugu..................	14	1	seq
......................................................................agcugccugguaaagagcuCu..................	3	1	seq
......................................................................agcCgccugguaaagagcugu..................	5	1	seq
......................................................................agcugcAugguaaagagcugu..................	10	1	seq
......................................................................agcugccugguUaagagcugu..................	3	1	seq
......................................................................agUugccugguaaagagcugu..................	1	1	seq
......................................................................agcugccuggGaaagagcugu..................	1	1	seq
......................................................................agcugccuUguaaagagcugu..................	2	1	seq
......................................................................agNugccugguaaagagcugu..................	1	1	seq
......................................................................agcuAccugguaaagagcugu..................	22	1	seq
......................................................................agcugccugguaaagagcuUu..................	2	1	seq
......................................................................agcugccugguaaagagUugu..................	1	1	seq
......................................................................agcugccGgguaaagagcugu..................	1	1	seq
......................................................................agcugccugguaaagagcugG..................	26	1	seq
......................................................................agcugUcugguaaagagcugu..................	2	1	seq
......................................................................agcugccugguaaagGgcugu..................	2	1	seq
......................................................................agcugccugguaaaAagcugu..................	1	1	seq
......................................................................agcugccugguaaagagAugu..................	3	1	seq
......................................................................agcGgccugguaaagagcugu..................	1	1	seq
......................................................................Ggcugccugguaaagagcugu..................	164	1	seq
......................................................................agcugAcugguaaagagcugu..................	5	1	seq
......................................................................aAcugccugguaaagagcugu..................	4	1	seq
......................................................................agcugccugguaaagagcAgu..................	4	1	seq
......................................................................agcugccCgguaaagagcugu..................	3	1	seq
......................................................................agcugccugguaaagagcugC..................	4	1	seq
......................................................................Ngcugccugguaaagagcugu..................	3	1	seq
......................................................................agcugccuAguaaagagcugu..................	13	1	seq
......................................................................agcugcGugguaaagagcugu..................	2	1	seq
......................................................................agcugccugguaaagaCcugu..................	1	1	seq
```

```
                                    Star                                   Mature
cuccugugcucacgucuccugguugaggacaguacggcucuuuaucugguagacuugugcaaacgccccaagcugccugguaaagagcugucucgucgcaacccuucuc
......................................................................Cgcugccugguaaagagcugu..................	3	1	seq
......................................................................agcugccugguaaagagcGgu..................	2	1	seq
......................................................................agcugccugguaaaUagcugu..................	1	1	seq
......................................................................agcugccugguaaagagGugu..................	1	1	seq
......................................................................agcugccugguaaagagcuAu..................	1	1	seq
......................................................................agcugccAgguaaagagcugu..................	9	1	seq
......................................................................agcugccugguGaagagcugu..................	6	1	seq
......................................................................agcugccugguaaagUgcugu..................	2	1	seq
......................................................................agcugccugAuaaagagcugu..................	4	1	seq
......................................................................aCcugccugguaaagagcugu..................	1	1	seq
......................................................................agcugccugguaUagagcuguc.................	32	1	seq
......................................................................agcugccugguaaagUgcuguc.................	46	1	seq
......................................................................agcugccugAuaaagagcuguc.................	95	1	seq
......................................................................agcugccugCuaaagagcuguc.................	76	1	seq
......................................................................agcugccuggGaaagagcuguc.................	19	1	seq
......................................................................agcugGcugguaaagagcuguc.................	15	1	seq
......................................................................agcAgccugguaaagagcuguc.................	262	1	seq
......................................................................agNugccugguaaagagcuguc.................	1	1	seq
......................................................................aCcugccugguaaagagcuguc.................	34	1	seq
......................................................................agcugccugguaaagagcuguG.................	25	1	seq
......................................................................agcGgccugguaaagagcuguc.................	24	1	seq
......................................................................agcugccugguaaagagcugAc.................	164	1	seq
......................................................................agcugccugguaaagagcuguU.................	1763	1	seq
......................................................................aNcugccugguaaagagcuguc.................	13	1	seq
......................................................................agcuCccugguaaagagcuguc.................	33	1	seq
......................................................................agcugccuggCaaagagcuguc.................	78	1	seq
......................................................................agcuUccugguaaagagcuguc.................	34	1	seq
......................................................................agcugUcugguaaagagcuguc.................	68	1	seq
......................................................................agcugcUugguaaagagcuguc.................	116	1	seq
......................................................................agcugccugguaaagaUcuguc.................	25	1	seq
......................................................................agcugccugguaCagagcuguc.................	6	1	seq
......................................................................agcugccugguaaCgagcuguc.................	2	1	seq
......................................................................agcugccugguaaUgagcuguc.................	47	1	seq
......................................................................agcugccugguaaagagcuUuc.................	17	1	seq
......................................................................agcugccAgguaaagagcuguc.................	212	1	seq
......................................................................agcugccugguaaaUagcuguc.................	17	1	seq
......................................................................agcugccugguaaagagUuguc.................	28	1	seq
......................................................................Ggcugccugguaaagagcuguc.................	4426	1	seq
......................................................................agcugccuAguaaagagcuguc.................	445	1	seq
......................................................................agcugccugguaaagagGuguc.................	26	1	seq
......................................................................agcugccCgguaaagagcuguc.................	60	1	seq
......................................................................agcugccugguaaagagcuAuc.................	68	1	seq
......................................................................agcugccugguaaagGgcuguc.................	71	1	seq
......................................................................agcugNcugguaaagagcuguc.................	2	1	seq
......................................................................agGugccugguaaagagcuguc.................	15	1	seq
......................................................................Ngcugccugguaaagagcuguc.................	158	1	seq
......................................................................agcugccugguCaagagcuguc.................	4	1	seq
......................................................................agcugccuCguaaagagcuguc.................	33	1	seq
......................................................................aAcugccugguaaagagcuguc.................	44	1	seq
......................................................................agcugccugguaaagagcugCc.................	52	1	seq
......................................................................agcugccugguaaagagcuguA.................	404	1	seq
......................................................................agcugccuggAaaagagcuguc.................	241	1	seq
......................................................................agcCgccugguaaagagcuguc.................	77	1	seq
......................................................................agcugcGugguaaagagcuguc.................	58	1	seq
......................................................................agcugccugguaaagagAuguc.................	60	1	seq
......................................................................agcugccugguaaagagcuCuc.................	35	1	seq
......................................................................agcugccGgguaaagagcuguc.................	10	1	seq
......................................................................agcugccugguaaagagcCguc.................	67	1	seq
......................................................................agcugccugUuaaagagcuguc.................	53	1	seq
......................................................................agcugccugguaaagagcGguc.................	28	1	seq
......................................................................agcugccugguaaagaAcuguc.................	29	1	seq
......................................................................agAugccugguaaagagcuguc.................	105	1	seq
......................................................................Ugcugccugguaaagagcuguc.................	31	1	seq
......................................................................agcugccugguaaagCgcuguc.................	8	1	seq
......................................................................agcuAccugguaaagagcuguc.................	74	1	seq
......................................................................aUcugccugguaaagagcuguc.................	66	1	seq
......................................................................agUugccugguaaagagcuguc.................	50	1	seq
......................................................................agcugccugguaaaAagcuguc.................	68	1	seq
......................................................................agcugcAugguaaagagcuguc.................	287	1	seq
......................................................................agcugccugguaaagagcuguc.................	466881	0	seq
```

```
                                  Star                              Mature
cuccugugcucacgucuccugguugaggacaguacggcucuuuaucugguagacuugugcaaacgccccaagcugccugguaaagagcugucucgucgcaacccuucuc
......................................................................agcugccugguUaagagcuguc.................  13     1  seq
......................................................................agcugccugguaGagagcuguc.................  101    1  seq
......................................................................agcugccuUguaaagagcuguc.................  26     1  seq
......................................................................Cgcugccugguaaagagcuguc.................  39     1  seq
......................................................................agcugccugguaaaCagcuguc.................  20     1  seq
......................................................................agcugccugguaaGgagcuguc.................  99     1  seq
......................................................................agcugccugguaaagagcugGc.................  27     1  seq
......................................................................agcugAcugguaaagagcuguc.................  237    1  seq
......................................................................agcugccugguGaagagcuguc.................  106    1  seq
......................................................................agcugccugguaaagaCcuguc.................  9      1  seq
......................................................................agcugccugguaaagagcAguc.................  90     1  seq
......................................................................agcugcUugguaaagagcugucu................  5      1  seq
......................................................................agAugccugguaaagagcugucu................  6      1  seq
......................................................................Cgcugccugguaaagagcugucu................  1      1  seq
......................................................................agcugccugguaaagagcAgucu................  6      1  seq
......................................................................agcugccugguaaUgagcugucu................  3      1  seq
......................................................................agcuUccugguaaagagcugucu................  1      1  seq
......................................................................agcugccugAuaaagagcugucu................  3      1  seq
......................................................................agcugccuggGaaagagcugucu................  2      1  seq
......................................................................agcugcGugguaaagagcugucu................  2      1  seq
......................................................................agcugccuCguaaagagcugucu................  3      1  seq
......................................................................agcugccugguUaagagcugucu................  1      1  seq
......................................................................agcuAccugguaaagagcugucu................  6      1  seq
......................................................................agcugccugguaaagagcuguAu................  13     1  seq
......................................................................agcugccugguaaagagcugucN................  1      1  seq
......................................................................agcugccugguaaagagcuguGu................  4      1  seq
......................................................................agcAgccugguaaagagcugucu................  24     1  seq
......................................................................agcugccugguaaagagGugucu................  2      1  seq
......................................................................agcugccuUguaaagagcugucu................  3      1  seq
......................................................................agcugccugguaaagagcuUucu................  2      1  seq
......................................................................agcugUcugguaaagagcugucu................  5      1  seq
......................................................................agcugccugguaaagagcugucu................  28776  0  seq
......................................................................agcugccugguaaagagAugucu................  2      1  seq
......................................................................agcugccugguaaagagUugucu................  1      1  seq
......................................................................agcGgccugguaaagagcugucu................  2      1  seq
......................................................................agcugccugguaUagagcugucu................  1      1  seq
......................................................................agcugccugguGaagagcugucu................  9      1  seq
......................................................................agcugccugguaaGgagcugucu................  6      1  seq
......................................................................agcugccugguaaagagcugAcu................  8      1  seq
......................................................................agcugccCgguaaagagcugucu................  2      1  seq
......................................................................agcugcAugguaaagagcugucu................  17     1  seq
......................................................................aUcugccugguaaagagcugucu................  4      1  seq
......................................................................agcugccugguaaagagcugGcu................  4      1  seq
......................................................................agcugccugguaaagagcuguUu................  33     1  seq
......................................................................agcugccAgguaaagagcugucu................  8      1  seq
......................................................................agcugccugUuaaagagcugucu................  2      1  seq
......................................................................agcugccuggCaaagagcugucu................  4      1  seq
......................................................................agcugccugguaaagCgcugucu................  2      1  seq
......................................................................agcugccugguaaagaAcugucu................  6      1  seq
......................................................................agcugccugguaaagagcCgucu................  27     1  seq
......................................................................agcugccugguaaagUgcugucu................  1      1  seq
......................................................................agcuCccugguaaagagcugucu................  1      1  seq
......................................................................Ugcugccugguaaagagcugucu................  4      1  seq
......................................................................agcugccugCuaaagagcugucu................  3      1  seq
......................................................................agcugccugguaaagGgcugucu................  4      1  seq
......................................................................aAcugccugguaaagagcugucu................  2      1  seq
......................................................................agcugccugguaaagagcuAucu................  1      1  seq
......................................................................Ngcugccugguaaagagcugucu................  10     1  seq
......................................................................agcugccugguaaagagcuCucu................  1      1  seq
......................................................................agcugccugguaaaAagcugucu................  3      1  seq
......................................................................agcCgccugguaaagagcugucu................  1      1  seq
......................................................................agcugccuggAaaagagcugucu................  19     1  seq
......................................................................agcugccugguaaagagcGgucu................  1      1  seq
......................................................................agcugccugguaaagagcuNucu................  1      1  seq
......................................................................agcugccugguaaagagcugucG................  22     1  seq
......................................................................aCcugccugguaaagagcugucu................  1      1  seq
......................................................................agcugAcugguaaagagcugucu................  22     1  seq
......................................................................agUugccugguaaagagcugucu................  2      1  seq
......................................................................agcugccugguaGagagcugucu................  17     1  seq
......................................................................agcugccugguaaagagcugCcu................  4      1  seq
```

```
                                  Star                                Mature
cuccugugcucacgucuccugguugaggacaguacggcucuuuaucugguagacuugugcaaacgccccaagcugccugguaaagagcugucucgucgcaacccuucuc
......................................................................agGugccugguaaagagcugucu................ 3     1  seq
......................................................................agcugccugguaaagaCcugucu................ 1     1  seq
......................................................................agcugccuAguaaagagcugucu................ 20    1  seq
......................................................................Ggcugccugguaaagagcugucu................ 264   1  seq
......................................................................agcugccugguaaagagcugucC................ 85    1  seq
......................................................................agcugccugguaaagagcugucA................ 1071  1  seq
......................................................................agcugccugguaaagagcugucuU............... 37    1  seq
......................................................................agcugccugguaaagagcugucuA............... 2     1  seq
......................................................................agcugccugguaaagagcugucAc............... 1     1  seq
......................................................................agcugccugguaaagagcugucuG............... 3     1  seq
.......................................................................gcugccugguaaagagcu.................... 1     0  seq
.......................................................................gcugccugguaaagagcug................... 1     0  seq
.......................................................................gcugccuUguaaagagcugu.................. 1     1  seq
.......................................................................gcugccugguaaaUagcugu.................. 1     1  seq
.......................................................................gcugccAgguaaagagcugu.................. 1     1  seq
.......................................................................gcugccugguaaagagcugu.................. 82    0  seq
.......................................................................gcAgccugguaaagagcugu.................. 2     1  seq
.......................................................................gcugccugguaaagagUuguc................. 1     1  seq
.......................................................................gcAgccugguaaagagcuguc................. 18    1  seq
.......................................................................gcugccGgguaaagagcuguc................. 1     1  seq
.......................................................................gcugccugguaaaAagcuguc................. 1     1  seq
.......................................................................gcugccuAguaaagagcuguc................. 1     1  seq
.......................................................................gcuAccugguaaagagcuguc................. 1     1  seq
.......................................................................gcugccuUguaaagagcuguc................. 1     1  seq
.......................................................................gcugccugguaaagagcuguU................. 2     1  seq
.......................................................................gcugccugguaaagagcuguA................. 2     1  seq
.......................................................................gcuUccugguaaagagcuguc................. 1     1  seq
.......................................................................gcugccugguaaagagcuguc................. 883   0  seq
.......................................................................gAugccugguaaagagcuguc................. 1     1  seq
.......................................................................gcugccugguaaagagcAguc................. 3     1  seq
.......................................................................gcugccAgguaaagagcuguc................. 12    1  seq
.......................................................................gcugccugguaaagagcugucC................ 1     1  seq
.......................................................................gcugccugguaaagagcugucu................ 388   0  seq
.......................................................................gcugccugguaaagagcugucA................ 2     1  seq
.......................................................................gcAgccugguaaagagcugucu................ 3     1  seq
.......................................................................Ucugccugguaaagagcugucu................ 1     1  seq
.......................................................................gcugccugguaaGgagcugucu................ 1     1  seq
.......................................................................gcugccugguaaagagcuguAu................ 1     1  seq
........................................................................cugccugguaaagagcuguc................. 14    0  seq
........................................................................cugccugguaaagagcuAucu................ 1     1  seq
........................................................................cugccugguaaagagcugucu................ 102   0  seq
.........................................................................ugccugguaaagagcugu.................. 1     0  seq
.........................................................................ugccugguaaagagcuguc................. 46    0  seq
.........................................................................ugccugguaaagagcugucu................ 4     0  seq
..........................................................................gccugguaaagagcuguc................. 18    0  seq
..........................................................................gccugguaaagagcugucu................ 9     0  seq
```
